# Antibacterial Effect of Sodium Hypochlorite and EDTA in Combination with High-Purity Nisin on an Endodontic-like Biofilm Model

**DOI:** 10.3390/antibiotics10091141

**Published:** 2021-09-21

**Authors:** Ericka T. Pinheiro, Lamprini Karygianni, Thomas Attin, Thomas Thurnheer

**Affiliations:** 1 Department of Dentistry, School of Dentistry, University of São Paulo, São Paulo 01000-000, Brazil; 2 Clinic of Conservative and Preventive Dentistry, Center of Dental Medicine, University of Zurich, CH-8032 Zürich, Switzerland; lamprini.karygianni@zzm.uzh.ch (L.K.); thomas.attin@zzm.uzh.ch (T.A.); thomas.thurnheer@zzm.uzh.ch (T.T.)

**Keywords:** nisin, sodium hypochlorite, EDTA, endodontic treatment, antibiofilm agents, oral biofilm model

## Abstract

Antimicrobial peptides have been proposed as antibiofilm agents. Therefore, we evaluated the effect of endodontic irrigants combined or not with the antimicrobial peptide nisin against an endodontic biofilm model composed of eleven bacterial species. Biofilms were grown on hydroxyapatite discs for 3, 15 and 21 days and treated with 1.5% sodium hypochlorite (NaOCl) or 17% EDTA followed by high-purity nisin (nisin ZP) or saline for 5 min each. Differences between groups were tested by two-way ANOVA and Tukey’s multiple comparisons test (*p* < 0.05). Treatment with 1.5% NaOCl completely eliminated 3-d and 15-d biofilms but did not eradicate 21-d biofilms. Treatment with 1.5% NaOCl and 17% EDTA was equally effective against 21-d biofilms, showing 5-log and 4-log cell reduction, respectively, compared to the untreated control (9 log_10_, *p* < 0.05). No significant difference was found between 1.5% NaOCl + nisin ZP and 1.5% NaOCl in 21-d biofilms (*p* > 0.05). Likewise, no significant difference was found between 17% EDTA + nisin ZP and 17% EDTA treatments (*p* > 0.05). In conclusion, 1.5% NaOCl or 17% EDTA were effective strategies to combat mature biofilms. The additional use of nisin did not improve the activity of conventional irrigants against multispecies biofilms.

## 1. Introduction

Apical periodontitis is a chronic inflammatory disease of the apical periodontium caused by polymicrobial infections organized as biofilms in root canals with necrotic pulp [1]. Intraradicular biofilms adhered to dentin walls are removed mainly by the mechanical action of instruments and irrigation with antimicrobial solutions [2]. However, as endodontic instruments cannot touch all dentin walls and current antimicrobial solutions cannot completely eliminate biofilms, most teeth still remain infected after root canal preparation [2]. Therefore, new strategies to remove and kill endodontic biofilms are needed.

Antimicrobial peptides have shown promising results as antibiofilm agents in medical microbiology since they have improved the antibiofilm activity of commonly used antimicrobial agents [3]. Nisin is a cationic antimicrobial peptide produced by lactic acid bacteria that interacts with anionic biofilm surfaces by causing disruption to the bacterial cell membrane [3]. The high-purity form of nisin (>95% purity) is more potent than the low-content nisin (2.5% purity) and has been considered a potential therapeutic agent to prevent oral biofilm formation or treat established oral biofilms [4,5]. High-purity nisin has been shown to be effective in preventing biofilm formation and decreasing cell viability in pre-established biofilms, especially those of Gram-positive species [6]. However, it showed low activity against an endodontic biofilm model, which mainly comprised Gram-negative anaerobic species [7]. It has been suggested that the outer membrane of Gram-negative organisms could prevent the antimicrobial peptide from reaching its target (lipid II) at the inner membrane. To circumvent this problem, the association of nisin with ethylene diamine tetra-acetic acid (EDTA) has been proposed [8].

EDTA is a cation chelator capable of destabilizing the outer membrane of Gram-negative bacteria, which may enhance the activity of other antimicrobials [8,9,10,11]. In addition, it is able to reduce the strength of the biofilm matrix by sequestering cations, thereby increasing the detachment of bacterial cells from the biofilm [8,9,10,11]. Given its antibiofilm activity, EDTA has been proposed as an alternative agent against pathogens of medical interest [8,9,10,11]. In dentistry, EDTA is commonly used to remove inorganic components of the smear layer produced during root canal preparation [12]. In the field of endodontics, EDTA has been primarily tested against *Enterococcus faecalis* biofilms [13,14,15]. Thus, a deeper analysis of its action against complex endodontic biofilms is necessary.

Considering that nisin is more effective against biofilms of Gram-positive species and that EDTA exerts its antimicrobial activity by dispersing biofilms and destabilizing the cell membrane, we hypothesized that the combined treatment of nisin and EDTA would substantially increase the effectiveness of both solutions against multispecies biofilms. Additionally, the synergistic effect of high-purity nisin and sodium hypochlorite (NaOCl), the gold standard solution for root canal irrigation, was investigated. Antibiofilm strategies were tested against an endodontic biofilm model of different ages (3, 15 and 21 days) in order to observe bacterial susceptibility during biofilm maturation.

## 2. Results

**Figure 1:**
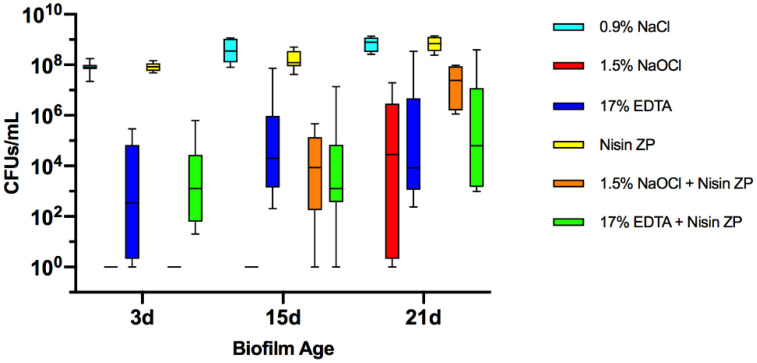
Boxplots demonstrating cell counts after 5 min treatments with 1.5% sodium hypochlorite (NaOCl); 17% EDTA; high-purity nisin (nisin ZP) and their associations on multispecies biofilms grown on hydroxyapatite disks for 3, 15 and 21 days. The internal line represents the median; the whiskers indicate minimum and maximum. Data derived from three independent experiments, each represented in triplicate biofilm cultures (*n* = 9).

Multispecies biofilms of 3, 15 and 21 days were treated with 0.9% saline (NaCl), 1.5% NaOCl, 17% EDTA, high-purity nisin (nisin ZP) and combinations of 1.5% NaOCl or 17% EDTA with nisin ZP. Colony-forming unit (CFU) counts in biofilms after treatment are shown in Figure 1. No bacterial growth was observed after 1.5% NaOCl treatment in 3- and 15-day biofilms. In contrast, the number of viable cells after 1.5% NaOCl treatment was much higher in 21-day biofilms than in younger biofilms (*p* < 0.01). Nevertheless, treatment with 1.5% NaOCl achieved 5-log cell reduction compared to the control in 21-day biofilms (*p* < 0.01). Similarly, the 17% EDTA treatment achieved 4-log cell reduction compared to saline (*p* < 0.02) in 21-day biofilms. Interestingly, no significant difference was found between 17% EDTA and 1.5% NaOCl groups in 21-day biofilms (*p* > 0.05). Nisin ZP was not effective in reducing bacteria in all biofilms, regardless of age. The 17% EDTA + nisin ZP group showed better antimicrobial activity than nisin ZP (*p* < 0.01). However, no significant differences were found between 17% EDTA + nisin ZP and 17% EDTA treatments in all tested biofilms (*p* > 0.05). Likewise, no significant differences were found between 1.5% NaOCl + nisin ZP and 1.5% NaOCl in 21-day biofilms (*p* > 0.05).

**Figure 2:**
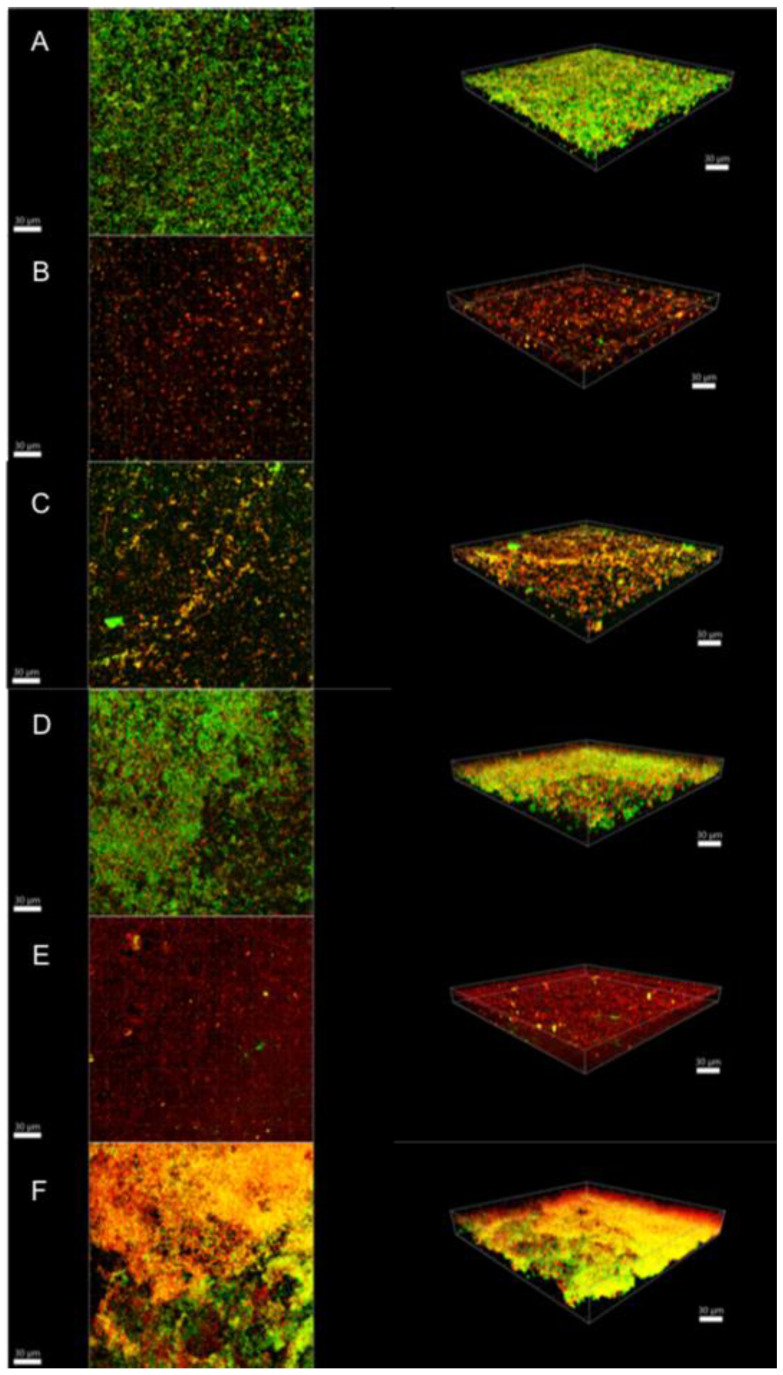
Confocal laser scanning microscopy images of 3-day biofilms. (**A**) Saline; (**B**) 1.5% NaOCl; (**C**) 17% EDTA; (**D**) high-purity nisin; (**E**) 1.5% NaOCl + high-purity nisin and (**F**) 17% EDTA + high-purity nisin. The biofilms were stained using the LIVE/DEAD Viability Kit; live cells appear green and dead cells red. Images represent the 3D reconstructions of the biofilms using IMARIS 7.6.5 software (orthogonal and perspective views from left to right). Scale bar 30 μm.

**Figure 3:**
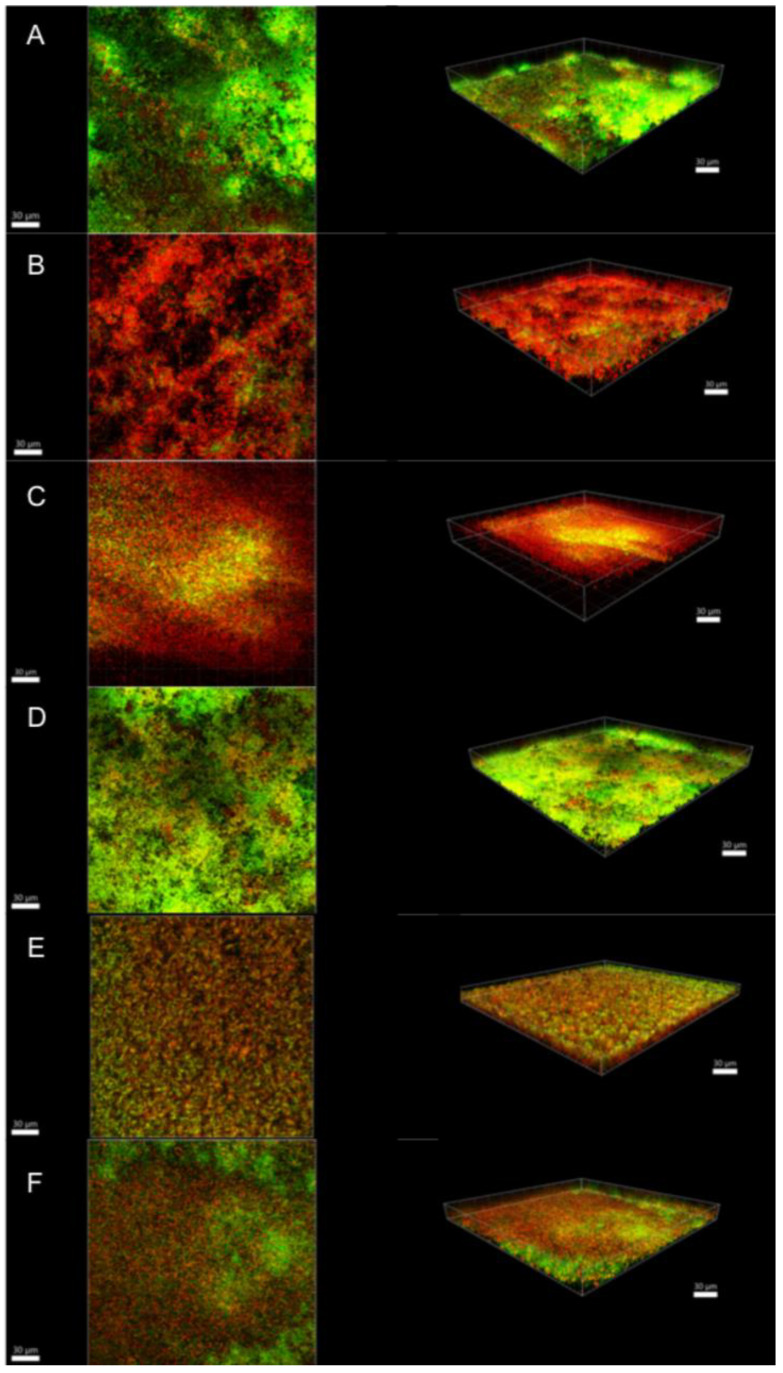
Confocal laser scanning microscopy images of 15-day biofilms. (**A**) Saline; (**B**) 1.5% NaOCl; (**C**) 17% EDTA; (**D**) high-purity nisin; (**E**) 1.5% NaOCl + high-purity nisin; and (**F**) 17% EDTA + high-purity nisin. The biofilms were stained using the LIVE/DEAD Viability Kit; live cells appear green and dead cells red. Images represent the 3D reconstructions of the biofilms using IMARIS 7.6.5 software (orthogonal and perspective views from left to right). Scale bar 30 μm.

**Figure 4:**
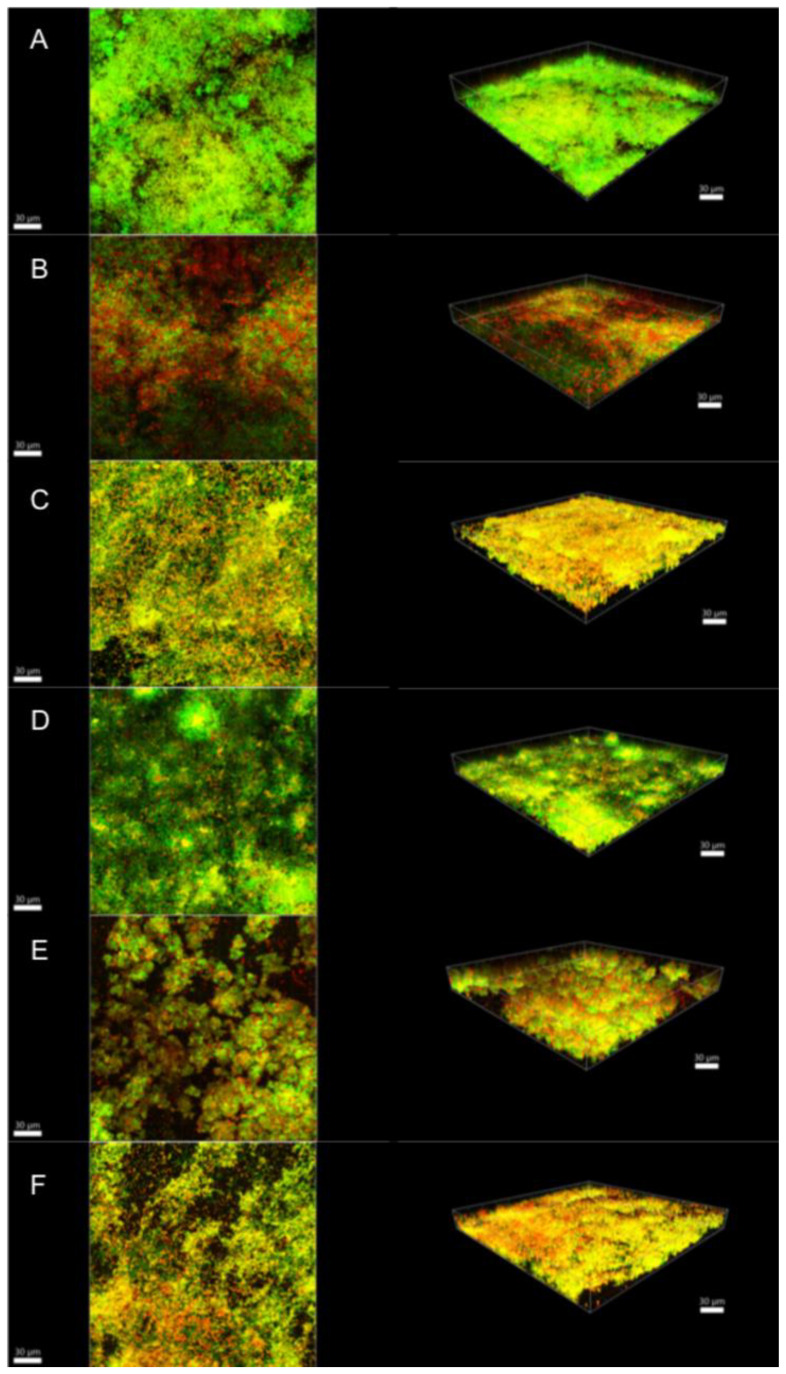
Confocal laser scanning microscopy images of 21-day biofilms. (**A**) saline; (**B**) 1.5% NaOCl; (**C**) 17% EDTA; (**D**) high-purity nisin; (**E**) 1.5% NaOCl + high-purity nisin and (**F**) 17% EDTA + high-purity nisin. The biofilms were stained using the LIVE/DEAD Viability Kit; live cells appear green and dead cells red. Images represent the 3D reconstructions of the biofilms using IMARIS 7.6.5 software (orthogonal and perspective views from left to right). Scale bar 30 μm.

Illustrative series of confocal laser scanning microscopy (CLSM) images of biofilms at different ages are shown in Figure 2, Figure 3 and Figure 4. These representative CLSM images reflect the findings of our culture analyses. After three days, the negative control (saline-treated biofilm) showed a dense biofilm where most of the cells seemed to be viable (stained green; Figure 2A). Treatment with 1.5% NaOCl or 1.5% NaOCl + high purity nisin (Figure 2B,E, respectively) resulted in a less dense biofilm where all the cells seemed to be dead (stained red). Figure 2C showed that treatment with 17% EDTA was less effective than with NaOCl as viable cells could still be detected. On the other hand, nisin ZP treatment showed no effect at all (Figure 2D), and the biofilm looked similar to the negative control (Figure 2A). In biofilms treated with 17% EDTA + nisin ZP (Figure 2F), yellow-stained cells predominated. Yellow cells may imply a transitional state from viable to dead or may also represent differences between bacterial species, as the amount of dye in the cells also depends on the intrinsic characteristics of the species. Comparable results were found for 15-day-old biofilms, although these biofilms were much denser and thicker, as expected (Figure 3). However, in the 21-day-old biofilms, the different treatments were obviously less effective (Figure 4). The biofilms seemed to be even more dense than in the younger biofilms, but in all treatments, the proportion of viable cells (green) and cells that were in transition (yellow) increased, whereas the proportion of dead cells (red) decreased.

## 3. Discussion

This laboratory study investigated the synergistic effect of high-purity nisin and 1.5% NaOCl or 17% EDTA using an endodontic-like biofilm model at different ages. Sodium hypochlorite (NaOCl) and EDTA promoted a significant bacterial reduction in 21-day-old biofilms. However, biofilms were not completely killed after using conventional endodontic irrigants after a 5-minute treatment. In turn, nisin did not improve the antibiofilm effects of 1.5% NaOCl or 17% EDTA. The number of bacteria in older biofilms was usually higher than in younger ones after antimicrobial treatment. This finding was consistent with previous reports showing that the level of maturation had an impact on the resistance of biofilms to endodontic disinfecting agents [16,17].

An eleven-species “endodontic-like” biofilm model, which included both Gram-negative and Gram-positive species commonly found in endodontic infections, was used in the present study. This biofilm model was previously validated on hydroxyapatite and dentin discs, which had a similar bacterial composition [18]. In this model, the endodontic pathogens were successfully incorporated into an established in vitro multispecies biofilm model, also known as the “Zurich” biofilm model [18]. The advantages of this biofilm model are standardization, reproducibility, easy interpretation and a community response pattern, which can provide greater resistance to disinfecting agents than a single microorganism [19]. On the other hand, the direct contact of irrigants with biofilms formed in the hydroxyapatite disks does not represent the clinical reality, where difficult-to-reach areas are expected due to the root canal anatomy [19]. Therefore, in vitro biofilm studies should be interpreted with caution, as they may provide more satisfactory results than those found under clinical conditions of endodontic treatment.

Sodium hypochlorite (NaOCl) is the most widely used solution for root canal irrigation due to its antimicrobial activity and capacity to dissolve necrotic pulp [12,20]. The properties of NaOCl depend on concentration and time, but so does its toxicity to apical tissues. Therefore, lower concentrations of NaOCl, used in greater volume, frequency and contact time, have been recommended for root canal disinfection [12,20]. The NaOCl concentration and contact time tested in the present study were chosen based on the clinical protocol recommended by the American Association of Endodontics (AAE) for regenerative endodontic procedures [21]. As mechanical cleaning is limited in these cases, the first steps for root canal disinfection are based on short-time irrigation (5 min) with a large volume of 1.5% NaOCl (20 mL), followed by irrigation with saline or EDTA. Although bacterial levels were reduced after 1.5% NaOCl, residual bacteria remained viable in the treated biofilms. This finding is in line with a previous clinical study showing that all root canals remained positive for bacteria after NaOCl irrigation during regenerative endodontic procedures [22]. This fact points to the need to seek new strategies for the disinfection of root canals in teeth with necrotic pulp and immature apex.

In the present study, we showed that the combined use of 1.5% NaOCl and high-purity nisin did not improve the NaOCl activity against mature polymicrobial biofilms. This finding is in contrast with previous studies showing a synergistic effect of nisin and 0.5% or 1% NaOCl against single-species biofilms of *Enterococcus faecalis* (2 days old) [6]. Differences between the studies may be related to the concentration of antimicrobials and the biofilm model, among other factors. In the present biofilm model, *E. faecalis* was integrated with ten other different bacterial species, allowing for complex bacterial interactions that assumingly have led to increased antimicrobial resistance in the community [18]. Future studies using mature polymicrobial biofilms should be performed in order to evaluate the possible synergy effect of nisin and lower NaOCl concentrations.

EDTA is a chelator agent commonly used to clean root canals by removing dentin particles from the smear layer formed during mechanical instrumentation [12]. A surprising finding of the present study was the bacterial reduction promoted by 17% EDTA in mature biofilms, which was similar to that achieved by the 1.5% NaOCl treatment. The antibiofilm activity of EDTA can be explained by its ability to detach cells in the biofilm, which occurs due to the destabilization of the biofilm matrix promoted by cation sequestration [8,9,10,11]. The cation-chelating action of EDTA can also interfere with bacterial growth by destabilizing the outer membrane of Gram-negative bacteria, which may explain its significant antibacterial activity against the tested biofilm model. However, as the identification of residual bacteria was not performed in the present study, no conclusive remarks can be drawn about the susceptibility of specific species to EDTA treatment. Future studies are needed to investigate the bacterial community that persists after treatment. For this purpose, the use of molecular methods could provide more information than culture-dependent methods due to their greater sensitivity and specificity.

The antibiofilm activity of EDTA is not a new concept [8,9,10,11,12,13]. However, as the antibacterial effect of EDTA on planktonic bacteria is limited, its antibiofilm activity may have been overlooked in the field of endodontics [12]. For instance, EDTA has been shown to have a weak bactericidal effect against *E. faecalis* planktonic cells but a strong effect on *E. faecalis* biofilms by reducing their biomass or integrity [14,15]. In fact, a previous study showed similar effects of 17% EDTA and 2% NaOCl on *E. faecalis* biofilms evaluated by CLSM [14]. It was consistent with our results, showing that there was no difference in bacterial counts after treatment of mature biofilms with either 17% EDTA or 1.5% NaOCl. The present study also showed a significant bacterial reduction after treatment with 17% EDTA in all biofilms tested. This finding is in line with a previous clinical study showing that EDTA was more effective than a saline solution in reducing root canal bacteria [23]. Furthermore, sequential irrigation with NaOCl and EDTA was shown to be more efficient in reducing bacteria and preventing biofilm regrowth than NaOCl alone [24]. These findings together reinforce the benefits of using EDTA as adjunctive therapy for biofilm removal from infected root canals.

In turn, the association of 17% EDTA and high-purity nisin was not superior to EDTA alone. This finding contrasts with a previous study showing a synergistic effect between lower concentrations of nisin and EDTA against *Salmonella* biofilms after 24 h of treatment [8]. In addition to drug concentrations and biofilm models, treatment time and duration may have impacted the study outcomes. In medicine, the exposure time of biofilms to EDTA is expected to be long, as they are generally used in formulations for topical use [8,9,10,11]. In contrast, the use of EDTA in endodontics is recommended for a short period of time, as prolonged exposure to demineralizing agents can weaken dentin [12]. Therefore, considering the concentration and contact time of EDTA in clinical conditions, the association of EDTA and nisin does not seem to have advantages over EDTA alone for the removal of endodontic biofilms.

## 4. Conclusions

This in vitro study highlighted the importance of using mature biofilms for the study of endodontic disinfecting agents. Using sodium hypochlorite (1.5% NaOCl) and 17% EDTA was an efficient strategy to control multispecies biofilms cultivated on hydroxyapatite discs. However, biofilms were not completely killed after short-term treatment. The additional use of nisin did not improve the activity of conventional irrigants against multispecies biofilms.

## 5. Materials and Methods

### 5.1. Biofilm Preparation

The “endodontic-like” biofilm model consisted of 11 bacterial species: *Actinomyces oris* OMZ 745, *Campylobacter rectus* OMZ 388 (ATCC 33238), *Enterococcus faecalis* OMZ 422 (ATCC 29212), *Fusobacterium nucleatum* OMZ 598 (KP-F2), *Parvimonas micra* OMZ 518 (ATCC 33270), *Porphyromonas gingivalis* OMZ 925 (ATCC 33277), *Prevotella intermedia* OMZ 278 (ATCC 25611), *Selenomonas sputigena* OMZ 527 (ATCC 35185), *Streptococcus oralis* OMZ 607 (SK 248), *Tannerella forsythia* OMZ 1132 (ATCC 43037) and *Veillonella dispar* OMZ 493 (ATCC 17748) [18]. Bacterial suspension was prepared using equal volumes and densities of each strain (optical density of 1.0 at 550 nm).

Biofilm preparation was performed as previously described [7,18]. Briefly, biofilms were grown in 24-well polystyrene cell-culture plates on hydroxyapatite (HA) discs (9-mm diameter; Clarkson Chromatography Products, South Williamsport, PA, USA). The HA disks were preconditioned in 1 mL of pasteurized and filter-sterilized saliva [25]. Then, the discs were covered with 1.6 mL of growth medium (modified fluid universal medium and saliva) and 200 μL of bacterial suspension [25]. Biofilms were incubated anaerobically at 37 °C for 3, 15 and 21 days. Fresh medium was provided daily during the incubation period.

### 5.2. Treatments

Disinfecting solutions were freshly prepared at the following concentrations: 1.5% NaOCl, 17% EDTA (Sigma-Aldrich, St Louis, MO, USA) and 200 μg/mL high-purity nisin (nisin ZP, Handary SA, Brussels, Belgium). The concentration of nisin ZP was chosen based on previous studies that tested its bactericidal activity and cytotoxicity to human oral cells [4,6]. Saline (0.9% NaCl) was used as a negative control. The solutions were used sequentially as follows: 1.5% NaOCl + saline, 17% EDTA + saline, nisin ZP + saline, 1.5% NaOCl + nisin ZP and 17% EDTA + nisin ZP. The discs were immersed in 1 mL of each solution for 5 min. Sequential treatment was used instead of mixed solutions due to the proteolytic activity of NaOCl, which can interfere with the activity of the peptide [26]. Furthermore, a saline wash was performed between the sequence solutions.

Three independent experiments were performed, including quadruplicates of each treatment. Three of the four discs were used to determine the total colony-forming units (CFUs), whereas one was randomly selected for confocal laser scanning microscopy (CLSM) analysis.

### 5.3. Estimation of Bacterial Counts

After treatment, the HA discs were washed twice in phosphate-buffered saline (PBS) and transferred to another tube with 1 mL of saline solution. Discs were then vortexed for 3 min, sonicated at 30 W for 10 s (Sonifier B-12, Branson Ultrasonics, Danbury, CT, USA) and vortexed again for 30 s. After serial dilutions, 50 µL of bacterial suspensions were plated onto Columbia blood agar plates using the Spiral System Model D (Spiral Systems Inc., Cincinnati, OH, USA). Plates were incubated anaerobically at 37 °C for 5 days, and colony-forming units (CFU) were counted. Differentiation of the species was achieved by observing the colonial morphology in conjunction with microscopic examination of cells from selected colonies.

### 5.4. Vital Staining and CLSM Analysis

Biofilm staining and CLSM analysis were performed as previously described [27]. Biofilms were stained using the LIVE/DEAD BacLight bacterial viability assay (Invitrogen, Zug, Switzerland). CLSM analysis was performed at three random positions on the disk using a Leica TCS SP5 (Leica Microsystems, Heidelberg, Germany). Image acquisition was performed in × 8 line average mode, and scans were recombined and processed using IMARIS 7.6.5 software (Bitplane, Zurich, Switzerland).

### 5.5. Statistical Analysis

Data from CFU counts were log transformed, and differences between the treatment groups were tested by two-way ANOVA followed by Tukey’s multiple comparisons test. A *p* value < 0.05 was considered to indicate a significant difference. Statistical analysis was performed using Prism v.8.4.3 statistical analysis software (GraphPad, La Jolla, CA, USA).

## Data Availability

Data are contained within the article.
